# Tumor Mutational Burden Predicting the Efficacy of Immune Checkpoint Inhibitors in Colorectal Cancer: A Systematic Review and Meta-Analysis

**DOI:** 10.3389/fimmu.2021.751407

**Published:** 2021-09-29

**Authors:** Yan Li, Yiqi Ma, Zijun Wu, Fanxin Zeng, Bin Song, Yanrong Zhang, Jinxing Li, Su Lui, Min Wu

**Affiliations:** ^1^ Huaxi MR Research Center (HMRRC), Department of Radiology, Functional and Molecular Imaging Key Laboratory of Sichuan Province, West China Hospital, Sichuan University, Chengdu, China; ^2^ Department of Clinic Medical Center, Dazhou Central Hospital, Dazhou, China; ^3^ Department of Radiology, School of Medicine, Stanford University, Stanford, CA, United States; ^4^ Department of Biomedical Engineering, Institute for Quantitative Health Science and Engineering (IQ), Michigan State University, East Lansing, MI, United States

**Keywords:** tumor mutation burden, immune checkpoint inhibitors, overall survival, objective response rate, colorectal cancer

## Abstract

**Objectives:**

For colorectal cancer patients, traditional biomarker deficient mismatch repair/microsatellite instability (dMMR/MSI) is an accurate predictor of immune checkpoint inhibitors (ICIs). Recent years, researchers considered tumor mutation burden (TMB) as another predictive biomarker which means the number of nonsynonymous mutations in cancer cells. Several studies have proven that TMB can evaluate the efficacy of ICI therapy in diverse types of cancer, especially in non-small cell lung cancer and melanoma. However, studies on the association between TMB and the response to ICI therapy in colorectal cancer alone are still lacking. In this study, we aim to verify the effect of TMB as a biomarker in predicting the efficacy of ICIs in colorectal cancer.

**Methods:**

We searched the PubMed and Ovid MEDLINE databases up to May 1, 2021 and screened studies for eligibility. Thirteen studies published from 2015 to 2021 with 5062 patients were included finally. We extracted and calculated hazard ratios (HRs) and odds ratios (ORs) of overall survival (OS) and objective response rates (ORRs) and their 95% confidence intervals (95% CIs). Pooled HR and OR were evaluated to compare OS and ORR between TMB-high and TMB-low groups in colorectal cancer patients. Meanwhile, we assessed heterogeneity with the *I* ^2^ statistic and p-values and performed publication bias assessments, sensitivity analyses, and subgroup analyses to search the cause of heterogeneity.

**Results:**

The TMB-high patient group had a longer OS than the TMB-low patient group (HR = 0.68, 95% CI: 0.51, 0.92, p = 0.013) among colorectal cancer patients receiving ICIs. In addition, the TMB-high patient group was superior in terms of ORR (OR = 19.25, 95% CI: 10.06, 36.82, p < 0.001) compared to the TMB-low patient group.

**Conclusions:**

In conclusion, this meta-analysis revealed that TMB can be used as a potential predictive biomarker of colorectal cancer patients receiving ICI therapy. Nevertheless, this finding is not stable enough. Therefore, many more randomized controlled trials are needed to prove that TMB is reliable enough to be used clinically to predict the efficacy of immunotherapy in colorectal cancer. And the most relevant biomarker remains to be determined when TMB high overlaps with other biomarkers like MSI and TILs.

## Introduction

Colorectal cancer (CRC) is a common intestinal malignancy. On the basis of the Global Cancer Epidemiological Statistics (GLOBOCAN2020) released by World Health Organization, it is estimated that in 2020, the number of new cases and deaths of colorectal cancer ranked second and third among all malignant tumors, respectively (1). With the approval of the first immune checkpoint inhibitor (ICI) (nivolumab) for colorectal cancer by the Food and Drug Administration (FDA) based on the data from the CHECKMATE142 trial in 2017, patients with stage IV colorectal cancer (deficient mismatch repair) can be treated with ICIs even if the tumor progresses after standard chemotherapy (2). A number of studies have shown that the status of deficient mismatch repair (dMMR)/microsatellite instability (MSI) can be used as a biomarker to identify patients who may benefit from immunotherapy (3–5). However, only 5% of patients with mCRC are dMMR/MSI-High and some of them do not respond to immunotherapy, which has limited clinical benefits. Meanwhile, there are still a small number of CRC patients with MSS who may benefit from ICI treatment (6). Therefore, it is necessary to look for more effective biomarkers to expand the CRC population responding to immunotherapy.

In addition to the status of dMMR/MSI, scientists have identified several biomarkers, including programmed cell death ligand 1 (PD-L1) expression, tumor infiltrating lymphocytes (TILs), POLE mutation and tumor mutation load (TMB) (7–10). TMB means the number of nonsynonymous mutations in cancer cells, which can be evaluated by next-generation sequencing (NGS) or whole-exome sequencing (WES). The higher the TMB is, the greater the type and number of neoantigens are produced by tumor cells, and the more likely it is to be recognized by the immune system. When ICIs activate their own antitumor immune response, the probability that these tumor cells will be killed is greater (11). One research combining 45 clinical studies and data from 103078 cancer patients found that TMB-high is an adverse prognostic factor for patients receiving non-immunotherapy, and a favorable factor for survival and efficacy for patients receiving immunotherapy, regardless of cancer type and TMB detection method (12). At present, the role of TMB as a predictor of immunotherapy has been confirmed in several specific types of cancer, such as non-small cell lung cancer (NSCLC) and melanoma (13–18), and preliminary studies have been carried out in patients with mCRC. Schrock AB et al. collected the response data of 22 MSI-HCRC patients treated with PD-1/L1 inhibitors and measured TMB by NGS. They found that the strongest correlation existed between TMB and objective response rate (ORR) and progression-free survival (PFS) by univariate analysis and multivariate analysis (19).

The study of TMB as a predictive biomarker has made great progress in NSCLC and melanoma (20, 21), but research on TMB in colorectal cancer has not yet been refined. The existing evidence is not sufficient to determine whether TMB can be used as a predictive biomarker of the immunotherapy in colorectal cancer. In view of the fact that the overall efficiency of immunotherapy in the field of colorectal cancer is not high, it is more urgent to select the dominant population. Here, we conducted this systematic review and meta-analysis to assess the predictive value of TMB on the effect of ICI treatment in patients with colorectal cancer based on the latest clinical evidence.

## Methods

This meta-analysis conformed to the Preferred Reporting Items for Systematic Reviews and Meta-Analyses (PRISMA) guidelines (22).

### Search Strategy, Study Selection, and Inclusion Criteria

We searched Ovid MEDLINE and PubMed databases up to May 1, 2021 with the following search terms: (mutational burden OR mutation burden OR mutational load OR mutation load OR TMB) AND (nivolumab OR pembrolizumab OR atezolizumab OR avelumab OR durvalumab OR ipilimumab OR tremelimumab OR immunotherapy OR PD-1 OR PD-L1 OR CTLA-4 OR immune checkpoint OR checkpoint blockade OR immune checkpoint inhibitors OR ICI OR ICIs OR immune checkpoint blockers OR ICB OR ICBs) AND (colon cancer OR colorectal cancer OR colorectal carcinoma OR colon carcinoma).

Studies were independently screened and reviewed by two investigators (Li and Ma), and differences were resolved through discussion and consensus. After removing duplicate reports, we first assessed the titles and abstracts of studies for eligibility following the present inclusion criteria: (1) studies had to assess the effect of TMB in predicting the outcomes of ICIs, such as anti-PD-1, anti-PD-L1, anti-CTLA-4, their combination, or other ICIs, in colorectal cancer. (2) Studies ought to provide the TMB-related hazard ratio (HR) and its 95% confidence interval (95% CI) of overall survival (OS) or odds ratio (OR) and its 95% CI of ORR. If above conditions were not met, studies had to give Kaplan–Meier curves or original OS or ORR data to generate calculable metrics. (3) Animal studies, reviews, comments, case reports, editorials, and conference abstracts were excluded, and studies were written in English. Studies deemed eligible were enrolled after full-text view.

### Data Extraction and Quality Assessment

We extracted the following data from the eligible studies: author, publication year, number of patients, type of ICI therapy, recruitment area of patients, proportion of dMMR/MSI, sample source, sequencing method of TMB, TMB cutoff value, TMB median value and its range, survival outcomes and HRs/ORs, and 95% CIs.

The types of eligible studies included randomized controlled trials (RCTs) and cohort studies. The quality assessment of cohort studies was performed *via* the Newcastle-Ottawa Scale (NOS) with scores of 0-9. Studies scores as 8-9 were recognized as high-quality studies, 5-7 indicated intermediate-quality studies and lower than 5 indicated low-quality studies and high risk of bias (23). Specifically, as we only included patients treated with durvalumab and tremelimumab in the RCT study, it was estimated by NOS as well (24).

### Statistical Analysis

We compared the OS and ORR between high and low TMB patient groups through HR and OR to verify the effect of TMB in predicting the efficiency of ICIs in colorectal cancer patients. For studies that provided Kaplan–Meier curves without HR of OS, we used Engauge Digitizer to extract survival data and the program files provided by Tierney et al. to calculate HRs and corresponding 95% CIs (25). For studies with original data, a Cox proportional hazards regression model was applied to calculate the OR of the ORR and corresponding 95% CIs by IBM SPSS Statistics 20.0. The summary HR or OR and 95% CI and *p*-values were estimated *via* STATA 15.0 (Stata Corporation, College Station, TX, USA). Heterogeneity was also evaluated by the *I^2^* statistic and *p*-value using STATA. High heterogeneity meta-analysis was conducted under a random-effects model, and low heterogeneity meta-analysis was performed under a fixed-effects model. Values of 25% < *I^2^* < 50%, 50% < *I^2^* <75% and *I^2^ <* 75% suggested low heterogeneity, intermediate heterogeneity and significant heterogeneity, respectively (26). When heterogeneity was large, publication bias assessments, sensitivity analyses, and subgroup analyses were conducted to investigate the cause of the heterogeneity. Funnel plots and Egger’s test were conducted as publication bias assessments methods to estimate whether adequate eligible studies were included in our study (27). Egger’s test was used to quantify the funnel plots, and *p* > 0.05 represented the absence of publication bias. Sensitivity analyses were used to estimate the impact of each study on the stability of the results. Subgroup analyses included subgroup by the number of patients, subgroup by recruitment area of patients, subgroup by TMB sequencing method and subgroup by TMB cutoff.

## Results

### Search Results, Study Characteristics and Quality Assessment

**Figure 1:**
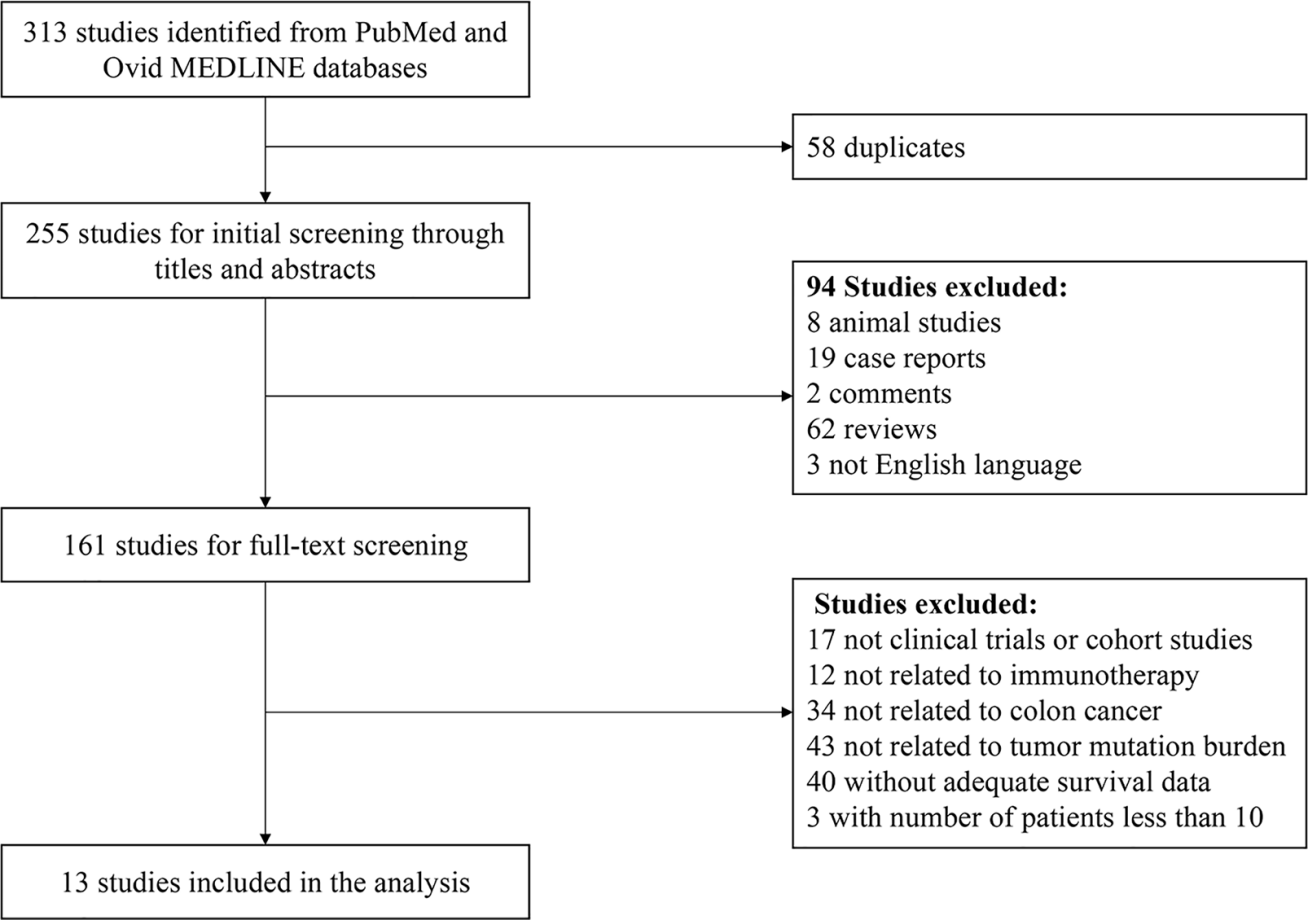
The PRISMA flowchart.

Based on our search strategy, a total of 255 studies were retrieved from the Ovid MEDLINE and PubMed databases after removing 58 duplicate studies. Animal studies, reviews, comments, case reports and non-English language studies were excluded through title and abstract screening. After removing studies not related to the topic, studies that were not clinical trials or cohort studies and studies without adequate survival data and sample size by full-text review, 13 studies published from 2015 to 2021 were finally included (**Figure 1**) (9, 19, 24, 28–37).

Study characteristics are shown in **Table 1**. A total of 5062 patient samples ranging from 15 to 2083 from each study were included in this meta-analysis. Among them, 8 studies were from Western countries, and 5 studies were from multiple areas. This analysis was composed of 1 RCT (24), 1 cohort study (28) and 11 retrospective cohort studies (9, 19, 29–37). Regarding the types of ICI therapy, 1 study used anti-PD-1(pembrolizumab) monotherapy, 1 study used anti-PD-L1 monotherapy, 3 studies adopted anti-PD-1/L1 therapy, and the remaining 8 studies used anti-CTLA-4 or anti-PD-1/L1 as monotherapy or in combination. Only 6 studies mentioned the proportion of dMMR/MSI mCRC. In these studies, WES and NGS were adopted to detect blood or tumor tissue samples. All but one of the studies adopted tumor tissue samples. A special study used blood samples for circulating cell-free DNA (cfDNA) (24). Different studies defined diverse TMB cutoffs. The survival data for 10 studies were expressed as OS, and 3 were expressed as ORR. According to the NOS, 9 studies that obtained a score of 7 were regarded as intermediate-quality, and 4 studies with scores of 8-9 were assessed as high-quality.

**Table 1:** Characters of included studies in the meta-analysis.

Reference	Number of Patients(High/Low TMB)	Area	Type of ICIs	dMMR/MSI	Sample Source	Sequencing Method	TMB Cutoff	Median TMB (Range)	Outcome	Score of NOS
Chen et al. 2020 (1, 24)	115(21/94)	Western	Anti-CTLA-4 and anti-PD-L1	0	Blood	NGS (GuardantOMNI)	28muts/Mb	15.3muts/Mb (0.96-85.4)	OS	7
Le et al. 2015 (1, 28)	15(NA)	Multiple areas	Anti-PD-1	60.0%	Tumor	WES	NA	771muts (5-4025)	OS	7
Lee et al. 2020 (1, 29)	63(NA)	western	Immune checkpoint inhibitors	NA	Tumor	NGS (MSK-IMPACT)	13.17muts/Mb	7.9muts/Mb (NA)	OS	7
Li et al. 2020 (1, 30)	403(NA)	Multiple areas	Immune checkpoint inhibitors	NA	Tumor	NA	NA	NA	OS	7
Lin et al. 2020 (1, 31)	109 (39/70)	western	Immune checkpoint inhibitors	NA	Tumor	NGS (MSK-IMPACT)	11muts/Mb	NA	OS	7
Peng et al. 2021 (1, 32)	398(NA)	western	Immune checkpoint inhibitors	NA	Tumor	NA	NA	9.95muts/Mb (0.05-188.32)	OS	7
Samstein et al. 2019 (1, 9)	110(22/88)	western	Immune checkpoint inhibitors	NA	Tumor	NGS (MSK-IMPACT)	52.2muts/Mb	7.90muts/Mb (0-203.64)	OS	8
Schrock et al. 2019 (1, 19)	22(13/9)	western	Anti-PD-1/L1	100%	Tumor	NGS	37-41muts/Mb	47.5muts/Mb (13-91)	ORR	7
Song et al. 2020 (1, 33)	109(87/22)	Multiple areas	Anti-PD-1/L1	NA	Tumor	NGS (MSK-IMPACT)	52.66muts/Mb	NA	OS	9
Valero et al. 2021 (1, 34)	50(43/7)	western	Anti-PD-1/L1	NA	Tumor	NGS (MSK-IMPACT)	10muts/Mb	NA	ORR	9
Yarchoan et al. 2019 (1, 35)	1141 (89/1052)	western	Anti-PD-1/L1	4.7%	Tumor	NGS (FoundationOne)	10muts/Mb	TMB-H:48.285 TMB-L:3.48	ORR	7
Zaidi et al. 2020 (1, 36)	2083(392/1691)	Multiple areas	Immune checkpoint inhibitors	14.7%	Tumor	NGS (AmpliSeq panel)	17muts/Mb	NA	OS	7
Zhou et al. 2021 (1, 37)	396(198/198)	Multiple areas	Immune checkpoint inhibitors	21%	Tumor	NA	96muts	96muts	OS	9

TMB, tumor mutational burden; ICIs, immune checkpoint inhibitors; CTLA-4, cytotoxic T lymphocyte-associated antigen 4; PD-L1, programmed death-ligand 1; PD-1, programmed cell death protein 1; dMMR, deficient mismatch repair; microsatellite instability, MSI; NGS, next-generation sequencing; WES, whole-exome sequencing; muts/Mb, mutations per megabase; muts mutations; OS, overall survival; ORR, objective response rate/overall response rate; NOS, Newcastle-Ottawa Scale; NA, not available.

### Main Results and Assessment of Heterogeneity

**Figure 2:**
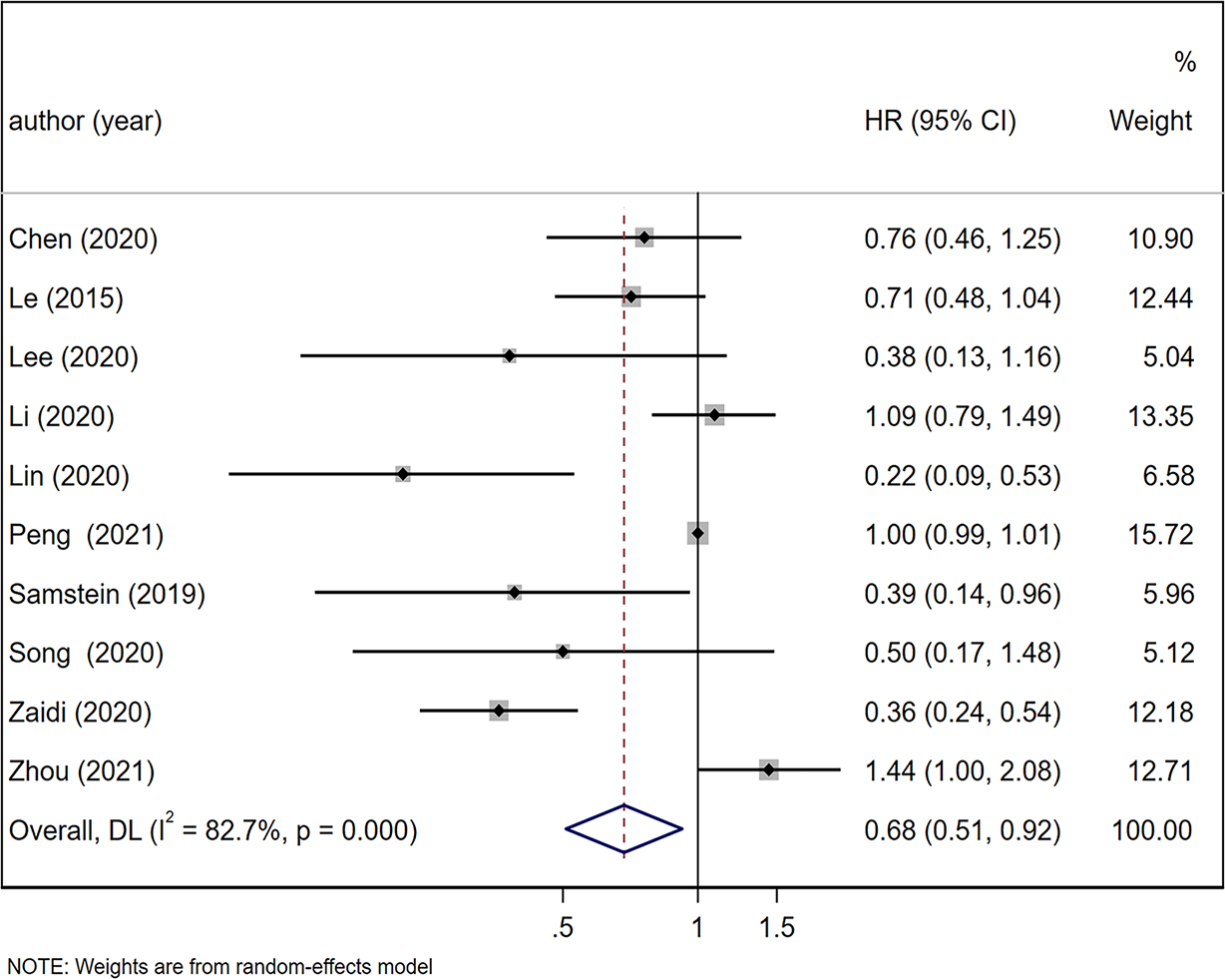
Forest plot of meta-analysis results of the association between overall survival and TMB. TMB, tumor mutation burden; HR, hazard ratio; CI, confidence interval.

**Figure 3:**
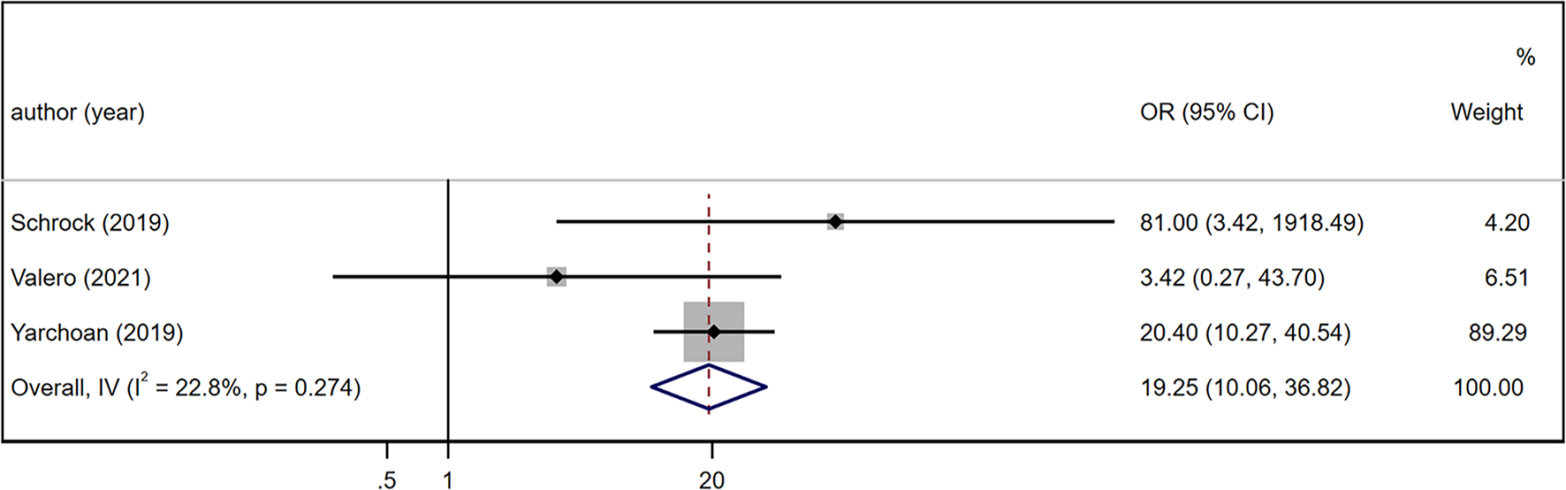
Forest plot of meta-analysis results of the association between objective response rate and TMB. TMB, tumor mutation burden; OR, odds ratio; CI, confidence interval.

Under a random-effects model, the summary HR of OS between the high and low TMB patient groups and the corresponding 95% CI were calculated based on 10 studies including 3849 patients (9, 24, 28–33, 36, 37). The results suggested that the OS of TMB-high patient group was longer than that of the TMB-low patient group (HR = 0.68, 95% CI: 0.51, 0.92, *p* = 0.013; **Figure 2**). The heterogeneity was significant in the comparison of OS in the TMB-high and TMB-low patient groups (*I^2^* = 82.7%, *p* < 0.001). Similarly, we assessed the summary OR of ORR in 3 studies with 1213 patients under a fixed-effects model (19, 34, 35). The TMB-high patient group was superior in terms of ORR (OR = 19.25, 95% CI: 10.06, 36.82, *p* < 0.001; **Figure 3**), and the heterogeneity was low (*I^2^* = 22.8%, *p* = 0.274).

### Publication Bias, Sensitivity Analyses and Subgroup Analyses

**Figure 4:**
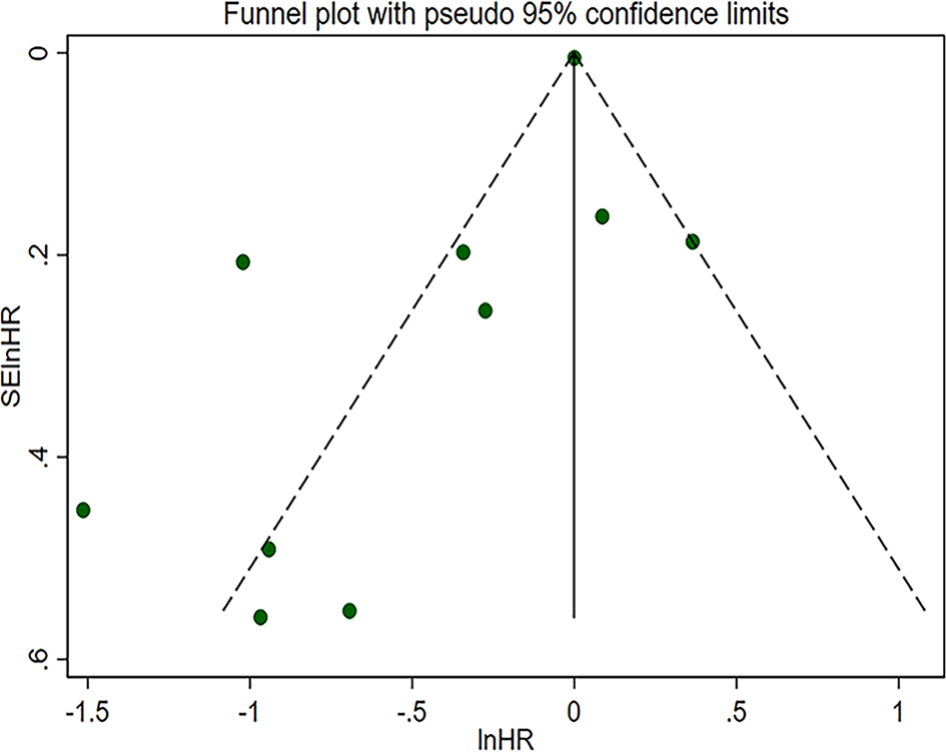
Funnel plot with pseudo 95% confidence limits of pooled overall survival. HR, hazard ratio.

To analyze the cause of high heterogeneity in the comparison of OS in TMB-high and low patient groups, we conducted publication bias, sensitivity analyses and subgroup analyses. The funnel plot is shown in **Figure 4**. Egger’s test (*p* = 0.053) quantitatively indicated that no publication bias existed. Sensitivity analyses suggested that after removing the study reported by Zaidi et al. (36), the combined 95% CI of the remaining 9 studies exceeded 1 [95% CI: 0.61, 1.03], indicating that TMB had no effect on OS, which was inconsistent with the results mentioned before, i.e., this study had a significant impact on the results. Therefore, this study was considered to have poor stability. After removal of this study, the *I^2^* decreased to 71.1%.

Subgroup analyses were performed on 4 aspects: number of patients, recruitment area of patients, sequencing method and TMB cutoff (**Table 2**). In the number of patients ≥ 100 subgroup, the OS of TMB-high patient group was greater than the TMB-low patient group (HR = 0.70, 95% CI: 0.50, 0.98, *p* = 0.041), but the heterogeneity was significant (*I^2^* = 84.8%, *p* < 0.001). In terms of the number of patients < 100 subgroup, the TMB-high and low patient groups had no statistically significant difference (HR = 0.65, 95% CI: 0.42, 1.00, *p* = 0.051) with low heterogeneity (*I^2^* = 10.3%, *p* = 0.291). In addition, no heterogeneity was noted between the two subgroups (*p* = 0.783). For the recruitment area of patients subgroup analysis, in the Western countries subgroup, the TMB-high patient group also showed superior OS (HR = 0.55, 95% CI: 0.31, 0.95, p = 0.034), while no significant difference was found in the multiple areas subgroup (HR = 0.76, 95% CI: 0.45, 1.27, *p* = 0.294). The heterogeneity of each subgroup was high (*I^2^* = 79.0%, *p* = 0.001) in the Western countries subgroup; and *I^2^* = 82.7%, *p* < 0.001 in the multiple areas subgroup). Similarly, no heterogeneity existed between them (*p* = 0.400). The sequencing method subgroup analysis only included studies using NGS for the detection of TMB and excluded the study (28) using WES. In the NGS (MSK-IMPACT) subgroup, the OS of the TMB-high patient group was greater than that of the TMB-low patient group (HR = 0.34, 95% CI: 0.21, 0.56, *p* < 0.001) and no heterogeneity was found in this subgroup (*I^2^* = 0.0%, *p* = 0.675). The NGS (non-MSK-IMPACT) subgroup included the NGS (GuardantOMNI) and NGS (AmpliSeq panel) methods and had a lower OS in the TMB-high patient group (HR = 0.52, 95% CI: 0.25, 1.07, *p* = 0.076) than in the NGS (MSK-IMPACT) subgroup, although the difference was not statistically significant in the NGS (non-MSK-IMPACT) subgroup. The heterogeneity was high in the NGS (non-MSK-IMPACT) subgroup (*I^2^* = 80.7%, *p* = 0.023) and no heterogeneity was found between the two subgroups (*p* = 0.362). Furthermore, we conducted subgroup analysis according to TMB cutoff. In the TMB cutoff < 28 muts/Mb subgroup, a better OS was observed in the TMB-high patient group (HR = 0.34, 95% CI: 0.24, 0.48, *p* < 0.001). Besides, this subgroup showed a better response to ICIs in CRC patients with higher TMB than the TMB cutoff ≥ 28 muts/Mb subgroup (HR = 0.63, 95% CI: 0.42, 0.96, *p* = 0.029). No heterogeneity was observed in both the TMB cutoff < 28 muts/Mb subgroup (*I^2^* = 0.0%, *p* = 0.596) and the TMB cutoff ≥ 28 muts/Mb subgroup (*I^2^* = 0.0%, *p* = 0.434) and the heterogeneity was significant between the two subgroups (*p* = 0.021).

**Table 2:** Subgroup analysis of overall survival between TMB-high and TMB-low group.

Subgroup	Number of Study	HR [95% CI]	*p*-Value	I^2^	Heterogeneity between subgroups
Number of patients					
patients ≥ 100	8	0.70 [0.50,0.98]	0.041	84.8%	*p* = 0.783
patients < 100	2	0.65 [0.42, 1.00]	0.051	10.3%	
Recruitment area of patients					
Western	5	0.55 [0.31, 0.95]	0.034	79.0%	*p* = 0.400
Multiple areas	5	0.76 [0.45, 1.27]	0.294	86.3%	
Sequencing method					
NGS (MSK-IMPACT)	4	0.34 [0.21, 0.56]	<0.001	0.0%	*p* =0.362
NGS (non-MSK-IMPACT)	2	0.52 [0.25, 1.07]	0.076	80.7%	
TMB cutoff					
TMB cutoff ≥ 28 muts/Mb	3	0.63 [0.42, 0.96]	0.029	0.0%	*p* =0.021
TMB cutoff < 28 muts/Mb	3	0.34 [0.24, 0.48]	<0.001	0.0%	

NGS, next-generation sequencing; TMB, tumor mutational burden; muts/Mb, mutations per megabase; HR, hazard ratio; CI, confidence interval.

## Discussion

The results of our study confirmed that both the OS and ORR of the TMB-high patient group were better than those of the TMB-low patient group. That is, high TMB could indicate a better response to ICI treatment. In this study, for patients with high TMB, the risk of death was reduced by 32% and the ORR was 19.25 times higher than that of patients with low TMB. However, as shown in the results of heterogeneity analyses, heterogeneity was significant in the comparison of OS in the TMB-high and low patient groups. Based on results of publication bias analysis, no publication bias existed. The sensitivity analyses indicated that the study reported by Zaidi et al. had influence on the stability of the results (36). The heterogeneity was significantly reduced after this study was removed. The NOS quality assessment revealed that most articles were of medium quality for this meta-analysis including the study researched by Zaidi et al. (36). To further investigate the cause of high heterogeneity, we chose four aspects to conduct subgroup analyses. First, we performed subgroup analysis based on the number of patients as different sample size, especially small sample size, may cause bias in the results. The predictive value of TMB may vary among patient populations so we conducted another subgroup analysis according to the recruitment areas of patients. Obviously, TMB value of the same individual could be different depending on the sequencing method which was one of the causes of difference of predictive effect of TMB on the efficiency of ICIs. Moreover, TMB cutoffs differed among studies. To determine a relatively appropriate cutoff range, we performed the last subgroup analysis on the basis of TMB cutoff. As the results, it seemed that differences in the number of patients, recruitment areas of patients and sequencing method were not responsible for high heterogeneity while different TMB cutoffs may be one of the explanations of the high heterogeneity. When we excluded studies without information on sequencing methods, heterogeneity decreased significantly (*I^2^* = 50.5%, p = 0.059), and heterogeneity was further reduced after removal of the study reported by Zaidi et al. (*I^2^* = 37.8%, p = 0.154) (36). The lack of included RCTs may also be related to high heterogeneity.

TMB refers to the number of nonsynonymous mutations in somatic cells in a specific genomic region, which can indirectly reflect the ability and degree of tumors to produce neoantigens. A large number of studies have shown that TMB can predict the efficacy of immunotherapy for many kinds of tumors (9, 11, 38). The detection of TMB is affected by many factors, such as the sample quality, sample source, detection methods, and analysis methods. The conditions of TMB detection should be fully understood before clinical application. Early studies on TMB used the method of WES, which covered approximately 22000 genes in the coding region, accounting for 1% of the whole genome. Studies have found that TMB measured by WES is related to the clinical benefits of immunotherapy for a variety of tumors (28, 39). However, the clinical application of WES is limited because of its high cost, long detection time, complex data analysis and the need for fresh samples (40). With the development of NGS, the whole genome can be sequenced quickly with the ultrahigh throughput, scalability and speed of NGS, and some studies have described that there is a significant correlation between the TMB detected by WES and the targeted panel (38, 41, 42). Unfortunately, only one study using WES to detect TMB was included in our study, so it was impossible to compare the effects of these two sequencing methods on the results. At present, a number of targeted sequencing panels have been approved by the FDA, and four panels which we included were used in the study, including MSK-IMPAKT, FoundationOne, GuardantOMNI and a custom AmpliSeq panel (24, 35, 43). These panels differ in some key parameters, but the most basic requirement of TMB detection is using NGS large panels (or WES), and the recommended sequencing depth is also different. The minimum targeted sequencing depth recommended in some studies should be ≥ 200 or ≥ 500, but in principle, the coverage of targeted sequencing should not be < 1.0 Mb, so as to ensure the accuracy of reporting TMB and to provide sufficient information (44, 45). In view of the sample size, we report the difference in the immunotherapy effect of TMB detected by the NGS-based MSK-IMPAKT panel and non-MSK-IMPAKT panel. Subgroup analysis showed that compared with the non-MSK-IMPAKT panel, the detection of TMB by the MSK-IMPAKT panel was significantly associated with OS benefits in CRC patients with TMB-H, and there was no heterogeneity in the NGS-based MSK-IMPAKT subgroup. The high heterogeneity in the non-MSK-IMPAKT group is a normal phenomenon, which may be due to the inclusion of two different targeting sequencing panels (GuardantOMNI and the custom AmpliSeq), and the predictive effect of TMB measured by these two panels is not as good as that of the MSK-IMPAKT group, either alone or in combination. This result may be due to the inadequate studies we included, but it also illustrates the potential advantages of MSK-IMPAKT panels. More researches will be needed in the future to explore the possible impact of TMB detection by different sequencing panels on the results.

Another factor affecting TMB detection is the source of samples. Tissue TMB (tTMB) detection is most common in clinical practice. In 2019, Samstein et al. conducted targeted NGS (MSK-IMPACT) of tumor tissues from 1662 patients with 10 advanced cancers treated with ICIs, including CRC. The results revealed that higher tTMB was responsible for better OS, and in most patients, the higher the tTMB was, the better the response to ICI treatment. This study provides strong evidence for the applicability of tTMB detection to more types of cancers (9). When tissue cannot be obtained clinically or the amount of tissue is not sufficient for TMB detection, the efficacy of immunotherapy can be predicted by evaluating blood TMB (bTMB). We included a RCT that analyzed the bTMB of patients with advanced refractory colorectal cancer who received combined ICIs and found that MSS patients with bTMB ≥ 28 mut/Mb had the greatest OS benefit (24). Although we did not perform a subgroup analysis of the sample sources due to the limitation of sample size in this meta-analysis, studies have confirmed that there is a significant correlation between bTMB and tTMB in NSCLC (13). At present, the correlation between the two still lacks of strong evidence in the clinical application of CRC, but it provides a possibility for patients with difficulties in obtaining tissue samples to predict the efficacy of immunotherapy and dynamically monitor treatment changes by evaluating bTMB.

One of the key problems of TMB is to determine the predictive cutoff point for immunotherapy. At present, there is no accurate standard for determining the critical value of TMB in solid tumors (including colorectal cancer). The TMB level was significantly different in various cancer types, with a difference of more than 1,000 times, and it was also highly heterogeneous in different patients with the same cancer type; for example, in malignant melanoma and lung cancer, the level of TMB of different patients varies from 0.1 to 100 mut/Mb (46). In 2020, the FDA approved anti-PD-1 therapy for any type of solid tumor with TMB ≥ 10 mut/Mb based on Keynote158 trial data (47). Although studies have confirmed that patients with TMB ≥ 10 mut/Mb have generally higher response rates to ICI treatment in many tumors (34), the predictive value of the TMB universal threshold is limited due to differences among various tumors, and it should be recommended to use the same threshold screening strategy with various tumors. A study published in 2019 by Memorial Sloan-Kettering Cancer Center on the prediction of TMB in the efficacy of tumor immunotherapy set the highest 20% TMB level in each histology as the cutoff value. The study found that in all patients, higher TMB was associated with better OS (HR 0.52; p=1.6×10^-6^), which is by far the largest cohort of patients receiving ICI treatment and may be a strategy for TMB threshold screening in multiple cancers (9). In our study, the difference of TMB cutoff point was still large, which may be due to the different detection methods, calculation methods and tumor heterogeneity (48–50). For example, Schrock et al. applied log-rank statistics to determine the optimal cut-point of TMB-H (19), Samstein et al. considered the top 20% of TMB in CRC patients as TMB-H group (9), while Valero et al. used the general threshold of TMB ≥ 10 approved by FDA (34), i.e. Because of the different TMB threshold screening strategies included in this study, it may be another reason for the heterogeneity of the results. In addition, in our study, the results of the subgroup analysis based on the TMB cutoff revealed that when the TMB cutoff < 28 muts/Mb, patients with higher TMB could have a better response to ICI therapy. Nevertheless, the optimal TMB cutoff point for CRC still needs to be determined through a large number of prospective clinical trials.

In theory, the more genetic mutations in cancer patients, the more neoantigens the cancer cells produce, and the more likely they are to be recognized by immune cells. There is a positive correlation between TMB and neoantigens, which is an important factor indicating that TMB can be used as a biomarker of ICIs. In addition to TMB, several other biomarkers also play an important role in predicting the prognosis of colorectal cancer and the efficacy of ICI. It is reported that these biomarkers overlap in varying degrees in CRC (51). Although the expression of PD-L1 is considered to be a biomarker for predicting ICI therapy in many cancers (such as lung cancer), current studies have shown that the expression of PD-L1 has no predictive value in CRC patients treated with ICI, and the prognostic value of its overexpression varies with the status of MSI (2, 28, 52). TILs and immunoscore also showed a good performance on prognosis and predictive value (8, 53). Immunoscore analysis of CRC patients showed better prognostic value than MSI, and patients with high immunoscore had a significantly lower risk of recurrence (54). At present, the researches on the predictive value of POLE mutation in ICI therapy are limited. Wang et al. analyzed the frequency of POLE/POLD1 gene mutation and its relationship with TMB and immunotherapy. The results showed that in the group of solid tumors treated with ICI, the total survival was better in patients with POLE or POLD1 mutation than that of non-carriers, and the TMB of patients with POLE or POLD1 gene mutation in a variety of tumors was significantly higher compared with that of non-carriers (including colorectal cancer). Among them, a small number of patients with POLE or POLD1 gene mutations were complicated with MSI-H, and the survival benefits were still significant even after removing these patients (55). In addition, in an analysis of the human cancer genome of 100,000 patients, it was found that the vast majority of MSI patients were TMB-high. The cooccurrence of these two phenotypes is highly dependent on the type of cancer. MSI-H and TMB-high always occur at the same time in gastrointestinal tumors, while TMB-high is quite common in melanoma, squamous cell carcinoma and lung cancer, but MSI-H is very rare (38). Fabrizio et al. also confirmed this view in CRC patients and found that nearly 3% of CRC patients with MSS were TMB-high (6). This finding may expand the CRC population that can benefit from ICI therapy. The relationship between the above predictors highlights the importance of finding a biomarker or a combination of biomarkers that are most relevant to the prediction of curative effect. The combination of TMB and MSI status may help to screen out CRC patients with pMMR/MSS who can benefit from ICI therapy, and eliminate patients with dMMR/MSI who may not benefit from ICI treatment. Patients with high expression of PD-L1 and TMB at the same time have better results with immunotherapy, and they are independent predictors (42). When POLE mutation and dMMR/MSI occurred at the same time, PFS and OS were significantly prolonged after ICI therapy (56). In the future, it is necessary to explore the most relevant biomarker or the best combination of biomarkers to predict the efficacy of immunotherapy in large-scale RCTs.

According to the expert opinions on immunotherapy for patients with CRC, immunotherapy or combination therapy are not recommended for pMMR/MSS CRC patients outside the trial conditions, while some combination therapies have shown potential activity in phase I/II studies for mCRC patients with pMMR/MSS (57, 58). Chen et al. also found that CRC patients of MSS with TMB-high who received combined immunotherapy had the greatest OS benefits (24). A phase II study is also under way to evaluate the efficacy of ICI in pMMR/MSS mCRC patients with high immunoscore (59). The above results need to be further verified by randomized III phase studies to determine the best immunotherapy strategy for pMMR/MSS mCRC patients.

This study is the first meta-analysis on the effect of TMB in the efficacy of ICI therapy in patients with colorectal cancer, which is of reference value for future studies on the association between TMB and immunotherapy and the clinical application of TMB in colorectal cancer patients. However, there are still some shortcomings in this study. Generally, TMB is still a controversial biomarker in clinical practice. There are still many problems in the standardization of TMB detection. More prospective studies are needed to verify how to select the TMB detection platform and targeted sequencing panel, how to determine a threshold screening strategy that can be applied to all kinds of tumors, whether there is a correlation between tTMB and bTMB in the field of colorectal cancer, and the independent predictive value of TMB in the Chinese population. On the other hand, studies on TMB as a predictive biomarker in immunotherapy of colorectal cancer are still insufficient. The interaction between TMB and other predictive biomarkers and the optimal prediction combination need to be verified by large scale randomized trials. Researches on TMB mainly focused on melanoma and NSCLC due to the higher tumor mutation load compared with colorectal cancer (38). This is considered to be the most prominent reason for the efficacy of ICI treatment in these cancers.

## Conclusions

Our meta-analysis demonstrated that CRC patients with high TMB can benefit from the ICI therapy, indicating that tumor mutation load can be used as another potential predictive biomarker for immunotherapy in CRC. However, this finding is not stable enough according to the results of sensitivity analysis. Hence, a large number of RCTs will be needed in the future to prove that TMB is a reliable biomarker for predicting the efficacy of immunotherapy for colorectal cancer. And the most relevant biomarker remains to be determined when TMB high overlaps with other biomarkers like MSI and TILs.

## Data Availability Statement

The original contributions presented in the study are included in the article. Further inquiries can be directed to the corresponding author.

## Author Contributions

YL and YM designed the study. YL, YM, ZW, FZ, and JL collected the data. YM, ZW, and YZ performed the statistical analysis. YL and YM wrote the first draft of the manuscript with support from other authors. BS, SL, and MW contributed to manuscript revision. All authors contributed to the article and approved the submitted version.

## Funding

This work was supported by the Natural Science Foundation of China (Grant 81501462); the Chengdu International Science and Technology Cooperation Funding (Grant 2019-GH02-00074-HZ); the 1·3·5 Project for Disciplines of Excellence-Clinical Research Incubation Project, West China Hospital, Sichuan University; and the Functional and Molecular Imaging Key Laboratory of Sichuan Province (Grant 2012JO0011).

## Conflict of Interest

The authors declare that the research was conducted in the absence of any commercial or financial relationships that could be construed as a potential conflict of interest.

## Publisher’s Note

All claims expressed in this article are solely those of the authors and do not necessarily represent those of their affiliated organizations, or those of the publisher, the editors and the reviewers. Any product that may be evaluated in this article, or claim that may be made by its manufacturer, is not guaranteed or endorsed by the publisher.
